# The impact of respiratory reactance in oscillometry on survival in patients with idiopathic pulmonary fibrosis

**DOI:** 10.1186/s12890-023-02776-y

**Published:** 2024-01-02

**Authors:** Tatsuru Ishikawa, Hirotaka Nishikiori, Yuki Mori, Keiko Fujino, Atsushi Saito, Mamoru Takahashi, Koji Kuronuma, Shiro Hinotsu, Hirofumi Chiba

**Affiliations:** 1 Department of Respiratory Medicine and Allergology, Sapporo Medical University School of Medicine, South-1, West-16 Chuo-Ku, Sapporo, Hokkaido 060-8556 Japan; 2 Department of Urology, Sapporo Medical University School of Medicine, South-1, West-16 Chuo-Ku, Sapporo, Hokkaido 060-8556 Japan; 3 Department of Biostatistics and Data Management, Sapporo Medical University School of Medicine, South-1, West-16 Chuo-Ku, Sapporo, Hokkaido 060-8556 Japan

**Keywords:** Oscillometry, Lung physiology, Interstitial lung disease (ILD), Idiopathic pulmonary fibrosis (IPF), Respiratory reactance

## Abstract

**Background:**

Idiopathic pulmonary fibrosis (IPF) is a progressive disease with a poor prognosis. Pulmonary function tests (PFTs) aid in evaluating the disease status of IPF. The clinical significance of oscillometry measurements in interstitial lung diseases has recently been reported. Our previous study showed that respiratory reactance (Xrs) measured by oscillometry reflected disease severity and predicted subsequent lung capacity decline in patients with IPF. However, the direct impact of Xrs on survival needs to be determined, and there are currently no reference values in oscillometry to predict prognosis. Therefore, this study aimed to investigate the association between oscillometry measurements, particularly Xrs, and survival in patients with IPF and to determine the cutoff values of Xrs that predict 3-year survival.

**Methods:**

We analyzed the relationship between the measured values of PFT and oscillometry derived from 178 patients with IPF. Univariate and multivariate Cox proportional hazards analyses were performed to investigate the relationships between clinical indices at the time of the first oscillometry and survival. We performed the time-dependent receiver operating characteristic (ROC) curve analysis to set the optimized cutoff values of Xrs for 3-year survival prediction. We examined the discriminating power of cutoff values of Xrs on survival using the Kaplan–Meier method and the log-rank test.

**Results:**

Xrs components, especially in the inspiratory phase (In), significantly correlated with the PFT values. In the multivariate analyses, Xrs (all of reactance at 5 Hz [X5], resonant frequency [Fres], and low-frequency reactance area [ALX] in the inspiratory phase) had a significant impact on survival (X5, *p* = 0.003; Fres, *p* = 0.016; ALX, *p* = 0.003) independent of age, sex, and other prognostic factors derived from the univariate analysis. The area under the ROC curve was 0.765, 0.759, and 0.766 for X5 In, Fres In, and ALX In, with cutoff values determined at − 0.98, 10.67, and 5.32, respectively. We found significant differences in survival after dividing patients using each of the cutoff values of Xrs.

**Conclusions:**

In patients with IPF, Xrs measured by oscillometry significantly impacted survival. We also determined the cutoff values of Xrs to discriminate patients with poor prognoses.

**Supplementary Information:**

The online version contains supplementary material available at 10.1186/s12890-023-02776-y.

## Background

Idiopathic pulmonary fibrosis (IPF) is the most common phenotype of chronic fibrosing interstitial lung disease (ILD) and shows a progressive course with a poor prognosis [1]. Forced vital capacity (FVC) and diffusing capacity of the lung for carbon monoxide (DLCO) are well-known as prognostic factors in patients with IPF [2–5]. A pulmonary function test (PFT) is also helpful in evaluating the disease status of IPF [6].

Oscillometry, also known as the forced oscillation technique, is a method of measuring the mechanical properties of the respiratory system (consisting of upper and intrathoracic airways, lung tissue, and chest wall) by the applying an oscillating pressure signal at the mouth during quiet tidal breathing [7]. Respiratory impedance measured by oscillometry is divided into respiratory resistance (Rrs) and reactance (Xrs). It is considered that Rrs reflects the caliber of the airway, and Xrs indicates the elasticity and inertia of the respiratory system [8]. There are various reports on the clinical significance of the values measured by oscillometry in patients with ILD [9–15]. Oscillometry and spirometry do not reflect the same physiological conditions of the respiratory tract system [8], but both are beneficial in the clinical practice of patients with ILD. Oscillometry can be performed with quiet tidal breathing, an advantage that even patients with severely deteriorated lung function can undergo with a reduced physical burden.

In our previous study [9], we found that Xrs showed significant correlations with values in PFT, reflected the severity of the disease, and predicted a subsequent decline in lung capacity in patients with IPF. However, the direct association of Xrs with survival should be determined. In addition, since there is no reference value in oscillometry for patients with IPF, we cannot predict a patient’s prognosis with values of oscillometry. Reference values in oscillometry for patients with IPF, which can distinguish the patient’s prognosis, should be determined.

In this investigation, we examined the association between values in oscillometry, especially Xrs, and survival in patients with IPF and determined the cutoff values of Xrs that predict 3-year survival.

## Methods

### Subjects

**Fig. 1:**
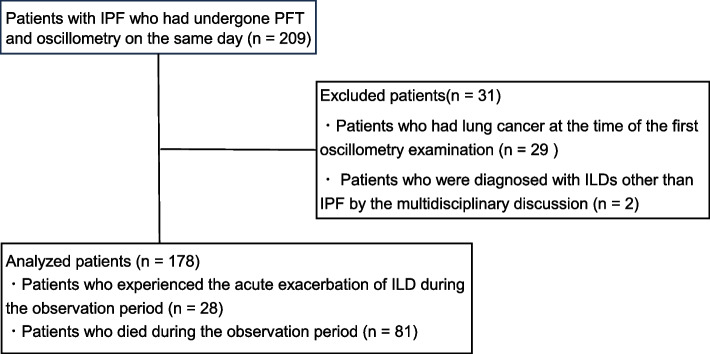
Flow diagram of study patients. ILD, interstitial lung disease; IPF, idiopathic pulmonary fibrosis; PFT, pulmonary function test

A total of 209 patients with IPF who had undergone PFT and oscillometry at the Sapporo Medical University Hospital from March 2012 to March 2020 were retrospectively investigated. We gathered the patient’s information which included age, sex, body mass index (BMI), smoking status, disease stage {Gender–Age–Physiology (GAP) stage [2]}, comorbidities, and the results of PFT and oscillometry at the time when the first oscillometry was performed from the patient’s clinical records. Survival data from the first performing date of oscillometry to March 31, 2022, were investigated. IPF was diagnosed by a multidisciplinary discussion based on the 2018 American Thoracic Society/European Respiratory Society/Japanese Respiratory Society/Latin American Thoracic Association statement [16]. No patient underwent lung resection and lung transplantation. Patients who had lung cancer at the time of the first oscillometry examination (*n* = 29) and who were diagnosed with ILDs other than IPF by the multidisciplinary discussion (*n* = 2) were excluded from this study. A total of 178 patients with IPF were finally included in this study (Fig. 1).

By performing Pearson’s chi-square test or Mann–Whitney U test, we compared patient background and PFT and oscillometry results between patients who survived for more than 3 years (good prognosis group) and those who died within 3 years (poor prognosis group) after oscillometry measurement. Survivors with an observation period of less than 3 years were excluded from the analysis (*n* = 17).

Because pulmonary emphysema coexistence might have an impact on the oscillometry results, we compared clinical indices between patients with combined pulmonary fibrosis and emphysema (CPFE) who met the criteria by Cottin et al. [17] (*n* = 30) and those without CPFE (*n* = 148). We also compared Xrs between patients with and without CPFE, adjusting for sex, age, FVC % predicted (%FVC) and DLCO % predicted (%DLCO) using analysis of covariance.

This study was conducted per the Declaration of Helsinki and approved by the institutional review board of the Sapporo Medical University Hospital (approval number 332–234; ref. May 16, 2022). Considering that this study is a retrospective observational research, the requirement of written informed consent from patients was waived.

### Measurement of respiratory impedance and pulmonary function

Respiratory impedance was measured using MostGraph-01 (Chest M.I. Co., Ltd, Japan), broadband oscillometry based on the standard recommendations [18]. Impulse oscillatory signals generated by a loudspeaker were applied to the respiratory system through the mouthpiece during a tidal breath of approximately 30 s. During the measurements, the subjects supported their cheeks to reduce upper airway shunting while sitting with their neck in a comfortable neutral posture. A nose clip was used to avoid nasal air leaks during oscillometry measurements. In this study, we measured and analyzed Rrs at 5 Hz (R5) and 20 Hz (R20), the difference between R5 and R20 (R5–R20), Xrs at 5 Hz (X5), resonant frequency (Fres), and low-frequency reactance area (ALX). Oscillatory indices were expressed as the mean value during a respiratory cycle (whole breath), the value in the expiratory phase (Ex), the value in the inspiratory phase (In), and the difference between the expiratory and inspiratory phases (Δ). Vital capacity (VC), FVC, forced expiratory volume in 1 s (FEV1), and DLCO were measured using a CHESTAC-8900 (Chest M.I. Co., Ltd, Japan) according to recommendations [19]. Oscillometry and PFT were performed on the same day, and oscillometry was performed before PFT.

### Relationship between the measured values of PFT and oscillometry

Correlations between the measured values of PFT and oscillometry were investigated using the Spearman's rank correlation coefficient. For patients who underwent several PFT and oscillometry evaluations, the initial measurement values were investigated.

### Relationship between clinical indices at baseline and survival

We performed the univariate and multivariate Cox proportional hazards analyses to investigate the relationships between clinical indices at the time of the first oscillometry and survival. The variables for the univariate analysis included age, sex, pack-years, BMI, comorbidities, and values of PFT and oscillometry. Statistically significant covariates in the univariate analysis were selected for the multivariate analysis. Variables that were reported to be clinically relevant to survival in patients with IPF (age, sex, %FVC, and %DLCO) [2] were also selected for the multivariate analysis.

### Determination of cutoff values of Xrs for 3-year survival prediction

As mentioned in the results, because Xrs, especially in the inspiratory phase, was strongly associated with survival, we performed the time-dependent receiver operating characteristic (ROC) curve analysis to set optimized cutoff values of Xrs (for each of X5 In, Fres In, ALX In) for a 3-year survival prediction. This time-dependent analysis was based on the Kaplan–Meier method. We created time-dependent ROC curves based on continuous values of Xrs (each of X5 In, Fres In, ALX In) and calculated the area under the curves (AUCs). The optimized cutoff values were set using the Youden index method. As described in the results, given that the Xrs values greatly correlated with %FVC, we also conducted the same analyses for %FVC as those for Xrs.

### Discriminating power of cutoff values of Xrs on survival

To examine the discriminating power of cutoff values of Xrs on survival, we compared patients’ estimated survival after classifying 178 patients into two groups of higher or lower than the cutoff values of Xrs using the Kaplan–Meier method and the log-rank test.

### Statistical analysis

The time-dependent ROC analysis was conducted using R software (version 4.3.1.), and other statistical analyses were performed using SPSS Statistics software (version 22). A* p*-value of < 0.05 was considered statistically significant.

## Results

### Patient characteristics

The baseline characteristics of the study population are shown in Table 1. The median age of the 178 participants was 69 (interquartile range [IQR], 64–75) years, with 140 men and 38 women. The median observation period was 48 (IQR, 29–72) months. DLCO could not be measured in 17 patients because of a severe cough or significant vital capacity loss. The median values of %FVC and %DLCO were 86.1% (IQR, 69.7%–101.7%) and 55.2% (IQR, 44.5%–66.3%), respectively. Regarding the GAP stage, we found stage I in 93 patients, stage II in 64 patients, and stage III in 21 patients.

**Table 1:** Baseline characteristics, results of the pulmonary function test and the oscillometry

	All analyzed patients (*n* = 178)	Good prognosis (*n* = 119)	Poor prognosis (*n* = 42)	*p*–value #
Age	69 (64 – 75)	68 (65 – 74)	69 (63 – 75)	0.958
Sex Men / Women	140 / 38	92 / 27	34 / 8	0.623
Outcome				
Survival / Death / Unknown	73 / 81 / 24			
GAP stage I / II / III	93 / 64 / 21	72 / 41 / 6	15 / 14 / 13	< 0.001*
BMI	23.5 (21.5 – 25.9)	23.5 (22.2 – 25.9)	22.7 (20.2 – 25.7)	0.102
Smoking				
Current or former / Never	146 / 32	98 / 21	34 / 8	0.839
Pack-years	35 (10 – 50)	35 (10 – 50)	35 (7 – 49)	0.692
Comorbidities				
Acute exacerbation of ILD^a^	4 (2.3)	1 (1.1)	3 (7.3)	0.024*
Cancer other than lung cancer	17 (10.6)	12 (12.8)	1 (2.4)	0.115
Hypertension	63 (54.8)	42 (44.7)	14 (34.1)	0.819
Hyperlipidemia	40 (29.0)	18 (19.1)	13 (31.7)	0.025*
Diabetes	40 (29.0)	29 (30.9)	7 (17.1)	0.303
Heart diseases	27 (17.9)	12 (12.8)	8 (19.5)	0.130
Arrhythmia	7 (4.1)	3 (3.2)	4 (9.8)	0.056
Pulmonary hypertension	7 (4.1)	5 (5.3)	1 (2.4)	0.592
Cerebrovascular diseases	14 (8.5)	8 (8.5)	5 (12.2)	0.289
Gastroesophageal reflux	14 (8.5)	11 (11.7)	3 (7.3)	0.678
Chronic renal diseases	17 (10.6)	10 (10.6)	7 (17.1)	0.090
Bronchial asthma	10 (6.0)	8 (8.5)	2 (4.9)	0.651
Indices of the PFT				
VC (L)	2.63 (2.18 – 3.39)	2.95 (2.39 – 3.56)	2.23 (1.76 – 2.59)	< 0.001^*^
%VC	87.8 (71.0 – 102.0)	96.5 (77.4 – 109.0)	70.3 (59.3 – 80.2)	< 0.001^*^
FVC (L)	2.60 (2.12 – 3.35)	2.89 (2.29 – 3.49)	2.12 (1.77 – 2.60)	< 0.001^*^
%FVC	86.1 (69.7 – 101.7)	95.7 (78.1 – 107.1)	68.4 (59.7 – 80.6)	< 0.001^*^
FEV1 (L)	2.15 (1.82 – 2.61)	2.27 (1.91 – 2.73)	1.91 (1.63 – 2.26)	< 0.001^*^
FEV1/FVC (%)	82.6 (77.4 – 87.6)	81.2 (76.7 – 84.6)	88.8 (83.5 – 93.4)	< 0.001^*^
DLCO (ml/min/mmHg)^b^	11.37 (9.36 – 14.91)	12.27 (10.01 – 15.61)	9.94 (8.54 – 11.99)	< 0.001^*^
%DLCO^b^	55.2 (44.5 – 66.3)	58.6 (48.5 – 71.8)	45.8 (35.9 – 57.0)	< 0.001^*^
Indices of the oscillometry				
R5 (cmH2O/L/s)				
Whole breath	2.70 (2.28 – 3.24)	2.73 (2.30 – 3.31)	2.78 (2.28 – 3.24)	0.817
Ex	2.84 (2.39 – 3.70)	2.86 (2.44 – 3.69)	2.83 (2.39 – 3.73)	0.703
In	2.46 (1.99 – 3.01)	2.47 (1.95 – 3.02)	2.42 (2.11 – 2.98)	0.774
ΔR5	0.37 (0.15 – 0.75)	0.38 (0.17 – 0.88)	0.39 (0.04 – 0.74)	0.872
R20 (cmH2O/L/s)				
Whole breath	2.07 (1.76 – 2.48)	2.15 (1.79 – 2.51)	1.94 (1.75 – 2.63)	0.424
Ex	2.13 (1.80 – 2.67)	2.22 (1.81 – 2.70)	2.04 (1.78 – 2.80)	0.330
In	1.99 (1.68 – 2.42)	1.99 (1.70 – 2.44)	1.88 (1.66 – 2.42)	0.494
ΔR20	0.12 (–0.05 – 0.40)	0.10 (–0.04 – 0.48)	0.16 (–0.11 – 0.39)	0.526
R5–R20 (cmH2O/L/s)				
Whole breath	0.62 (0.43 – 0.83)	0.60 (0.39 – 0.82)	0.62 (0.46 – 0.84)	0.474
Ex	0.76 (0.48 – 1.00)	0.75 (0.47 – 0.99)	0.81 (0.60 – 1.00)	0.586
In	0.47 (0.32 – 0.69)	0.47 (0.30 – 0.68)	0.49 (0.38 – 0.70)	0.376
ΔR5–R20	0.25 (0.08 – 0.43)	0.25 (0.10 – 0.45)	0.28 (0.11 – 0.42)	0.817
X5 (cmH2O/L/s)				
Whole breath	–0.87 (–1.16 to –0.48)	–0.75 (–1.04 to –0.40)	–1.14 (–1.66 to –0.97)	< 0.001^*^
Ex	–0.90 (–1.24 to –0.44)	–0.78 (–1.14 to –0.33)	–1.15 (–1.47 to –0.84)	< 0.001^*^
In	–0.82 (–1.17 to –0.50)	–0.73 (–0.98 to –0.37)	–1.18 (–1.87 to –0.83)	< 0.001^*^
ΔX5	0.04 (–0.12 – 0.20)	0.03 (–0.14 – 0.19)	0.07 (–0.05 – 0.25)	0.121
Fres (Hz)				
Whole breath	10.38 (7.88 – 12.34)	9.63 (7.46 – 11.17)	11.87 (10.38 – 14.30)	< 0.001^*^
Ex	10.16 (7.80 – 12.48)	9.61 (7.16 – 11.72)	11.82 (10.08 – 13.80)	< 0.001^*^
In	10.22 (8.20 – 12.09)	9.50 (7.57 – 11.32)	11.80 (10.22 – 14.83)	< 0.001^*^
ΔFres	–0.14 (–1.11 – 0.76)	–0.18 (–1.04 – 0.94)	–0.22 (–1.22 – 0.22)	0.281
ALX (cmH2O/L/s)				
Whole breath	3.71 (1.74 – 5.85)	3.06 (1.34 – 4.80)	5.62 (4.13 – 9.30)	< 0.001^*^
Ex	3.72 (1.57 – 6.01)	2.86 (1.07 – 5.37)	5.66 (3.50 – 8.42)	< 0.001^*^
In	3.43 (1.83 – 5.68)	2.95 (1.28 – 4.55)	5.84 (3.44 – 11.17)	< 0.001^*^
ΔALX	–0.24 (–0.97 – 0.62)	–0.13 (–0.72 – 0.67)	–0.62 (–1.53 – 0.38)	0.013^*^

Data are presented as median (interquartile range) or number of patients (percentage). #Differences between patients with a good prognosis and those with a poor prognosis

*ALX* Low-frequency reactance area, *BMI* Body mass index, *Δ* Difference between expiratory and inspiratory phases, *DLCO* Diffusing capacity of the lung for carbon monoxide, *%DLCO* Diffusing capacity of the lung for carbon monoxide (% predicted), *Ex* Expiratory phase, *FEV1* Forced expiratory volume in 1 s, *Fres* Resonant frequency, *FVC* Forced vital capacity, *%FVC* Forced vital capacity (% predicted), *GAP* Gender–Age–Physiology, *ILD* Interstitial lung disease, *In* Inspiratory phase, *PFT* Pulmonary function test, *R5* Resistance at 5 Hz, *R20* Resistance at 20 Hz, *R5–R20* Difference between R5 and R20, *VC* Vital capacity, *X5* Reactance at 5 Hz

^*^p-value < 0.05

^a^Before the first measurement of oscillometry

^b^DLCO was measured in 161 cases

Compared with the poor prognosis group, the good prognosis group showed a milder disease in GAP stage and better measurement values in the PFT and Xrs indices (higher X5 and lower Fres and ALX) in the oscillometry, with a lower rate of having acute exacerbation (AE) history and hyperlipidemia as comorbidities.

Furthermore, patients with CPFE had a higher tendency to exhibit a more preserved FVC and were more likely to be smokers and male than those without CPFE (Supplementary Table A.1). After adjusting for sex, age, %FVC, and %DLCO, no differences were observed in Xrs between patients with and without CPFE (Supplementary Table A.2).

### Correlations between the PFT and oscillometry values

The R5 (whole breath, Ex, In), R20 (whole breath, Ex, In), and R5–R20 (whole breath, Ex, In) showed significant negative correlations with the VC, FVC, and FEV1 (Table 2). The X5 (whole breath, Ex, In) showed significant positive correlations with the VC, %VC, FVC, %FVC, FEV1, DLCO, while the Fres (whole breath, Ex, In) and ALX (whole breath, Ex, In) showed significant negative correlations with the VC, %VC, FVC, %FVC, FEV1, DLCO (Table 2). In particular, the Xrs values in the inspiratory phase demonstrated strong correlations with the VC, %VC, FVC, %FVC and FEV1 (*r* = 0.6–0.7, *p* < 0.01). Additionally, X5 (whole breath, In) was positively correlated with %DLCO and negatively correlated with FEV1/FVC. Fres (whole breath, In) and ALX (whole breath, In) were negatively correlated with %DLCO and positively correlated with FEV1/FVC.

**Table 2:** Correlations between the pulmonary function test and oscillometry values

	VC	%VC	FVC	%FVC	FEV1	FEV1/FVC	^a^DLCO	^a^%DLCO
R5 Whole breath	–0.350^*^	–0.134	–0.349^*^	–0.123	–0.431^*^	–0.127	–0.110	0.007
R5 Ex	–0.321^*^	–0.084	–0.322^*^	–0.083	–0.408^*^	–0.132	–0.082	0.033
R5 In	–0.340^*^	–0.159^#^	–0.334^*^	–0.140	–0.411^*^	–0.133	–0.144	–0.035
ΔR5	–0.166^#^	0.028	–0.174^#^	–0.004	–0.240^*^	–0.099	–0.054	0.010
R20 Whole breath	–0.341^*^	–0.126	–0.340^*^	–0.111	–0.429^*^	–0.144	–0.065	0.035
R20 Ex	–0.336^*^	–0.090	–0.338^*^	–0.082	–0.430^*^	–0.143	–0.055	0.049
R20 In	–0.294^*^	–0.134	–0.288^*^	–0.108	–0.365^*^	–0.144	–0.069	0.013
ΔR20	–0.202^*^	0.002	–0.214^*^	–0.040	–0.276^*^	–0.077	–0.042	0.027
R5–R20 Whole breath	–0.276^*^	–0.146	–0.276^*^	–0.154^#^	–0.311^*^	–0.028	–0.144	–0.037
R5–R20 Ex	–0.209^*^	–0.079	–0.210^*^	–0.091	–0.243^*^	–0.032	–0.096	–0.002
R5–R20 In	–0.316^*^	–0.204^*^	–0.313^*^	–0.205^*^	–0.351^*^	–0.026	–0.213^*^	–0.109
ΔR5–R20	–0.017	0.086	–0.016	0.083	–0.059	–0.099	0.043	0.076
X5 Whole breath	0.687^*^	0.592^*^	0.669^*^	0.592^*^	0.663^*^	–0.245^*^	0.439^*^	0.329^*^
X5 Ex	0.568^*^	0.470^*^	0.553^*^	0.473^*^	0.579^*^	–0.146	0.310^*^	0.212^*^
X5 In	0.741^*^	0.673^*^	0.722^*^	0.669^*^	0.684^*^	–0.326^*^	0.505^*^	0.395^*^
ΔX5	–0.259^*^	–0.294^*^	–0.259^*^	–0.291^*^	–0.159^#^	0.326^*^	–0.216^*^	–0.221^*^
Fres Whole breath	–0.652^*^	–0.617^*^	–0.635^*^	–0.622^*^	–0.614^*^	0.285^*^	–0.426^*^	–0.315^*^
Fres Ex	–0.557^*^	–0.517^*^	–0.541^*^	–0.524^*^	–0.540^*^	0.215^*^	–0.322^*^	–0.218^*^
Fres In	–0.700^*^	–0.683^*^	–0.684^*^	–0.682^*^	–0.643^*^	0.341^*^	–0.494^*^	–0.386^*^
ΔFres	0.198^*^	0.226^*^	0.204^*^	0.227^*^	0.130	–0.238^*^	0.221^*^	0.235^*^
ALX Whole breath	–0.685^*^	–0.600^*^	–0.669^*^	–0.603^*^	–0.658^*^	0.263^*^	–0.434^*^	–0.321^*^
ALX Ex	–0.574^*^	–0.489^*^	–0.560^*^	–0.495^*^	–0.578^*^	0.171^#^	–0.317^*^	–0.216^*^
ALX In	–0.740^*^	–0.682^*^	–0.723^*^	–0.681^*^	–0.680^*^	0.342^*^	–0.510^*^	–0.396^*^
ΔALX	0.345^*^	0.371^*^	0.342^*^	0.360^*^	0.239^*^	–0.367^*^	0.249^*^	0.240^*^

The values in the table are the correlation coefficients

*ALX* Low-frequency reactance area, *Δ* Difference between expiratory and inspiratory phases, *DLCO* Diffusing capacity of the lung for carbon monoxide, *%DLCO* Diffusing capacity of the lung for carbon monoxide (% predicted), *Ex* Expiratory phase, *FEV1* Forced expiratory volume in 1 s, *Fres* Resonant frequency, *FVC* Forced vital capacity, *%FVC* Forced vital capacity (% predicted), *In* Inspiratory phase, *R5* Resistance at 5 Hz, *R20* Resistance at 20 Hz, *R5–R20* Difference between R5 and R20, *VC* vital capacity, *X5* Reactance at 5 Hz

^*^p-value < 0.01

^#^*p*-value < 0.05

^a^DLCO was measured in 161 cases

### Impact of baseline clinical indices on survival

During the observation period, 81 patients died. In the univariate analysis, all Xrs indices were significant predictive factors for survival (Table 3). The impact of Xrs was stronger in the inspiratory phase than in the expiratory phase. BMI, AE history, hyperlipidemia comorbidity, VC, %VC, FVC, %FVC, FEV1, FEV1/FVC, DLCO, and %DLCO were also significant predictive factors for survival (*p* < 0.05).

**Table 3:** Univariate Cox proportional-hazard analysis

	Hazard ratio	95% CI	*p*-value
Age	1.019	0.988–1.052	0.235
Sex	1.271	0.725–2.227	0.403
BMI	0.921	0.858–0.990	0.025^#^
Pack-years	0.998	0.991–1.006	0.611
Acute exacerbation of ILD^a^	6.094	2.205–16.840	< 0.001^*^
Cancer other than lung cancer	0.367	0.116–1.164	0.089
Hypertension	1.013	0.645–1.591	0.956
Hyperlipidemia	1.748	1.068–2.862	0.026^#^
Diabetes	0.827	0.489–1.398	0.478
Heart diseases	1.391	0.767–2.522	0.277
Arrhythmia	1.335	0.486–3.668	0.575
Pulmonary hypertension	0.287	0.040–2.070	0.216
Cerebrovascular diseases	1.008	0.406–2.502	0.986
Gastroesophageal reflux	0.886	0.385–2.037	0.775
Chronic renal diseases	1.587	0.792–3.177	0.193
Bronchial asthma	0.545	0.172–1.729	0.303
VC	0.453	0.339–0.606	< 0.001^*^
%VC	0.972	0.964–0.981	< 0.001^*^
FVC	0.446	0.330–0.601	< 0.001^*^
%FVC	0.974	0.965–0.982	< 0.001^*^
FEV1	0.456	0.307–0.678	< 0.001^*^
FEV1/FVC	1.089	1.055–1.123	< 0.001^*^
DLCO	0.837	0.779–0.899	< 0.001^*^
%DLCO	0.962	0.947–0.977	< 0.001^*^
R5 Whole breath	0.954	0.753–1.208	0.694
R5 Ex	0.940	0.775–1.141	0.534
R5 In	0.940	0.710–1.243	0.663
ΔR5	0.893	0.640–1.245	0.504
R20 Whole breath	0.803	0.564–1.142	0.222
R20 Ex	0.799	0.584–1.092	0.158
R20 In	0.759	0.518–1.113	0.159
ΔR20	0.794	0.455–1.386	0.418
R5–R20 Whole breath	1.276	0.822–1.981	0.278
R5–R20 Ex	1.089	0.777–1.527	0.628
R5–R20 In	1.826	0.997–3.343	0.051
ΔR5–R20	0.756	0.413–1.383	0.364
X5 Whole breath	0.458	0.344–0.610	< 0.001^*^
X5 Ex	0.653	0.522–0.817	< 0.001^*^
X5 In	0.322	0.232–0.447	< 0.001^*^
ΔX5	1.523	0.877–2.643	0.135
Fres Whole breath	1.276	1.172–1.390	< 0.001^*^
Fres Ex	1.176	1.097–1.261	< 0.001^*^
Fres In	1.326	1.215–1.447	< 0.001^*^
ΔFres	0.884	0.766–1.019	0.089
ALX Whole breath	1.127	1.078–1.178	< 0.001^*^
ALX Ex	1.061	1.025–1.098	0.001^*^
ALX In	1.194	1.137–1.254	< 0.001^*^
ΔALX	0.908	0.821–1.005	0.062

*ALX* Low-frequency reactance area, *BMI* Body mass index, *CI* Confidence interval, *Δ* Difference between expiratory and inspiratory phases, *DLCO* Diffusing capacity of the lung for carbon monoxide, *%DLCO* Diffusing capacity of the lung for carbon monoxide (% predicted), *Ex* Expiratory phase, *FEV1* Forced expiratory volume in 1 s; Fres, resonant frequency; FVC, forced vital capacity; %FVC, forced vital capacity (% predicted); ILD, interstitial lung disease; In, inspiratory phase; R5, resistance at 5 Hz, *R20* Resistance at 20 Hz R5–R20, difference between R5 and R20, *VC* Vital capacity, *X5* Reactance at 5 Hz

^*^p-value < 0.01

^#^*p*-value < 0.05

^a^Before the first measurement of oscillometry

In the multivariate analysis, we selected each Xrs index in the inspiratory phase as representative variables for Xrs. Age, sex, %FVC (represents VC, %VC, FVC, and %FVC), %DLCO (represents DLCO and %DLCO), FEV1 (represents FEV1 and FEV1/FVC), BMI, AE history, hyperlipidemia comorbidity, and each X5 In, Fres In, and ALX In were included as variables. All Xrs indices in the inspiratory phase were significant independent predictive factors for survival (Table 4).

**Table 4:** Multivariate Cox proportional-hazard analysis

	Hazard ratio	95% CI	*p*-value
X5 In	0.333	0.160–0.693	0.003^*^
Fres In	1.190	1.033–1.371	0.016^*^
ALX In	1.182	1.058–1.319	0.003^*^

Adjusted by age, sex, %FVC, %DLCO, FEV1, BMI, hyperlipidemia, and acute exacerbation history before the first measurement of oscillometry

*ALX* Low-frequency reactance area, *BMI* Body mass index, *CI* Confidence interval, *%DLCO* Diffusing capacity of the lung for carbon monoxide (% predicted), *FEV1* forced expiratory volume in 1 s; Fres, resonant frequency, *%FVC* Forced vital capacity (% predicted), *In* Inspiratory phase, *X5* Reactance at 5 Hz

^*^p-value < 0.05

### Cutoff values of Xrs for 3-year survival prediction

**Fig. 2:**
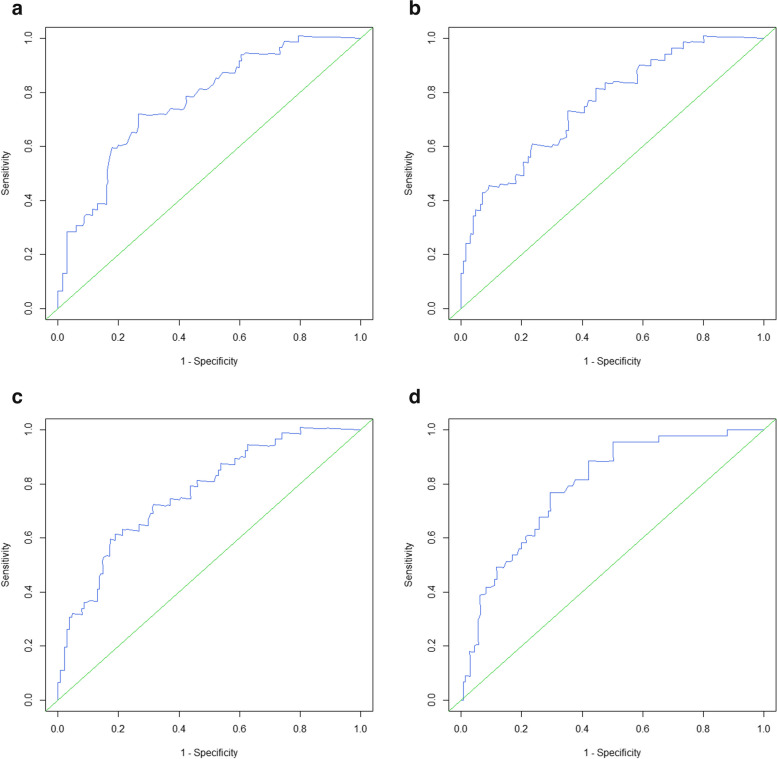
The time-dependent ROC curves of each Xrs index and %FVC for the 3-year survival prediction. X5 In (**a**), Fres In (**b**), ALX In (**c**), and %FVC (**d**). ALX, low-frequency reactance area; Fres, resonant frequency; %FVC, forced vital capacity (% predicted); In, inspiratory phase; X5, reactance at 5 Hz; Xrs, respiratory reactance

The time-dependent ROC curve analysis was conducted to evaluate the impact of Xrs in the inspiratory phase on 3-year survival and determine the cutoff values. The AUCs of the ROC curves were 0.765 for X5 In, 0.759 for Fres In, and 0.766 for ALX In (Fig. 2). In addition, the cutoff values were determined at − 0.98, 10.67, and 5.32 for X5 In, Fres In, and ALX In, respectively. The same analysis was conducted for %FVC. The AUC of the ROC curve for %FVC was 0.792, and the optimized cutoff value was 80.8 (Fig. 2).

### Discriminating power of cutoff values of Xrs on survival

**Fig. 3:**
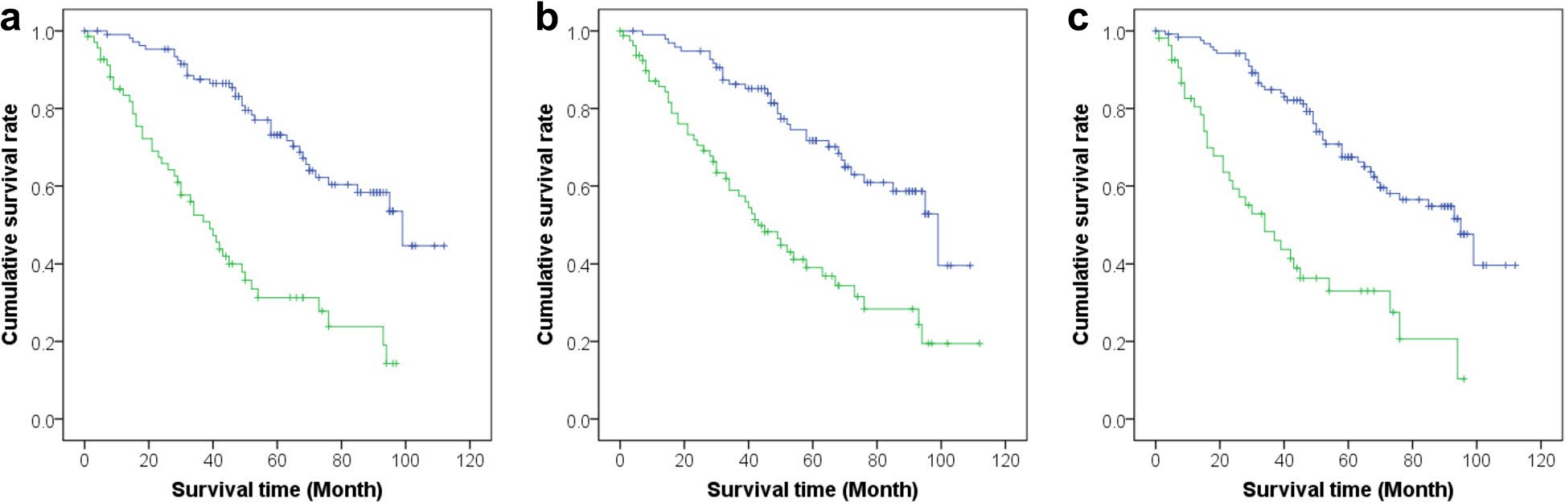
The Kaplan–Meier curves dividing patients with each optimized cutoff values of the respiratory reactance index. X5 In (**a**), Fres In (**b**), and ALX In (**c**). ALX, low-frequency reactance area; Fres, resonant frequency; In, inspiratory phase; X5, reactance at 5 Hz

The median survival time (MST) of the 178 patients was 73 (IQR, 34–not reached) months. We found significant differences in survival after dividing patients according to the cutoff values of Xrs (X5 In [≤ − 0.98 vs. >  − 0.98]: MST, 39 months vs. 99 months, *p* < 0.001; Fres In [≥ 10.67 vs. < 10.67]: MST, 43 months vs. 99 months, *p* < 0.001; ALX In [≥ 5.32 vs. < 5.32]: MST, 34 months vs. 95 months, *p* < 0.001) (Fig. 3).

## Discussion

IPF is a chronic progressive lung disease with a poor prognosis; however, the progression of the disease widely varies among individuals [1]. Various prognostic factors have been reported: age, sex, %FVC, and %DLCO [2–5], and the GAP stage that includes these variables are widely used to discriminate the predicted prognosis [2]. The distance in the 6-min walking test [20] and high serum levels of Krebs von den Lungen-6 [21], Surfactant protein-A and Surfactant protein-D [22], and neutrophil counts in bronchoalveolar lavage [23] have also been reported as prognostic factors for IPF; however, to the best of our knowledge, this is the first report describing the impact of respiratory impedance measured by the oscillometry on survival in patients with IPF.

In the Xrs, three indices are mainly measured: X5, which is Xrs at 5 Hz; Fres, which is the resonant frequency at point 0; and ALX, which is the low-frequency area (integral from X5 to Fres). In this study, we found particularly strong correlations between the values of Xrs in the inspiratory phase and VC, %VC, FVC, %FVC, and FEV1, with correlation coefficients of 0.6–0.7. These findings are consistent with our previous report [9]. Yamamoto et al. recently reported that the values of Xrs correlated with lung abnormalities on high-resolution computed tomography (HRCT), which were derived from fibrosis in patients with IPF using HRCT scores [10]. It is thought that Xrs, especially in the inspiratory phase, reflects restrictive impairment resulting from the loss of elasticity in the fibrotic lung in patients with IPF.

We previously reported lower X5 and higher Fres and ALX in patients with more advanced IPF, and these values worsened over time [9]. Wu et al. obtained similar results but used a different device [15]. In both the univariate and multivariate analyses, Xrs significantly affected survival. Importantly, over half of the participants were in GAP stage I, indicating that even in the early stage of IPF, Xrs indices are useful for predicting patients’ prognosis.

Reference values of MostGraph measures for middle-aged and elderly Japanese individuals who participated in annual health checkups were reported [24], but there is no reference value for patients with IPF. Although this study showed a significant impact of Xrs on survival in patients with IPF, how to utilize the obtained results of oscillometry in the actual clinical practice is a significant issue since there is no established reference value for Xrs, unlike for %FVC and %DLCO. The cutoff values of Xrs for prediction of the 3-year survival determined in this study would be helpful to discriminate the subsequent prognosis at the time of IPF diagnosis and at an appropriate time point during follow-up. Further large-scale studies are needed to prove the accuracy of the cutoff values.

Oscillometry is not a surrogate test for PFT, but it provides supplemental information. A study was conducted to develop regression equations to estimate VC and FVC using oscillometry indices in patients with asthma, chronic obstructive pulmonary disease, and ILD [14]; however, the accuracy of these equations needed to be improved. PFT requires breathing effort, and the measured values are thought to be affected by respiratory muscle strength. Conversely, oscillometric components can be measured with tidal breathing and are not affected by respiratory muscle strength. Oscillometry can be applied for patients with significantly reduced pulmonary function wherein PFT cannot be performed. Given that the number of patients with reduced pulmonary function included in this study was insufficient, we could not conduct a subset analysis. The prognostic discriminability among these patients should be elucidated in future studies. Among the components of oscillometry, Xrs is thought to reflect un-dissipative physical properties, such as lung elasticity and air inertia [8]. Considering these differences in measurement methods and the physical characteristics of Xrs, it can be inferred that VC (or FVC) measured by PFT and Xrs measured by oscillometry reflect different physiological states. In this study, we found that the indices of oscillometry reflected the prognosis in patients with IPF, and future studies which prove the further use of oscillometry in assessing pathophysiology and disease status in IPF and ILD, would increase the opportunities for examination and lead to the widespread use of oscillometry in patients with these diseases.

This study has several limitations. First, as a retrospective, single-center study in a tertiary hospital, there may be selection bias and estimated cutoff value in the ROC analysis also contains bias. The generalizability of the results to other facilities (such as community hospitals) needs to be demonstrated. Second, we did not investigate the details of IPF treatment, including the use of antifibrotic drugs, which have been reported to delay lung function deterioration and reduce the risk of AE [25, 26]. Therefore, the impact of treatment on patient outcomes needs to be fully assessed. Third, this study only included patients with IPF, and it is unclear whether the results can be extrapolated to patients with other fibrosing interstitial lung diseases. Finally, differences in measured values between oscillometry devices have been reported, and it is still being determined whether the results of this study can be applied to other oscillometry devices.

## Conclusions

In patients with IPF, Xrs measured by oscillometry was a significant independent predictive factor for survival in the multivariate analysis and a good discriminative factor for 3-year survival according to the time-dependent ROC analysis. We also determined the cutoff values of Xrs to discriminate patients with poor prognoses.

### Supplementary Information

**Additional file 1: Supplementary Table A.1.** Baseline characteristics, results of the pulmonary function test and the oscillometry. **Supplementary Table A.2.** Differences in Xrs between patients with and without CPFE adjusting for sex, age, %FVC, and %DLCO.

## Data Availability

The datasets used and/or analyzed during the current study are available from the corresponding author on reasonable request.

## Abbreviations

AE: Acute exacerbation
ALX: Low-frequency reactance area
AUC: Area under the curve
BMI: Body mass index
CPFE: Combined pulmonary fibrosis and emphysema
Δ: Difference between expiratory and inspiratory phases
DLCO: Diffusing capacity of the lung for carbon monoxide
%DLCO: Diffusing capacity of the lung for carbon monoxide (% predicted)
Ex: Expiratory phase
FEV1: Forced expiratory volume in 1 s
Fres: Resonant frequency
FVC: Forced vital capacity
%FVC: Forced vital capacity (% predicted)
GAP: Gender–Age–Physiology
HRCT: High-resolution computed tomography
IQR: Interquartile range
ILD: Interstitial lung disease
In: Inspiratory phase
IPF: Idiopathic pulmonary fibrosis
MST: Median survival time
PFT: Pulmonary function test
R5: Resistance at 5 Hz
R20: Resistance at 20 Hz
R5–R20: Difference between R5 and R20
ROC: Receiver operating characteristic
Rrs: Respiratory resistance
VC: Vital capacity
X5: Reactance at 5 Hz
Xrs: Respiratory reactance
