# Supplementary material for: The impact of respiratory reactance in oscillometry on survival in patients with idiopathic pulmonary fibrosis
Source: BMC Pulm Med. 2024 Jan 2;24:10. doi: 10.1186/s12890-023-02776-y (PMC10763674; doi:10.1186/s12890-023-02776-y)
Supplement: Supplementary file 1 — Additional file 1: Supplementary Table A.1. Baseline characteristics, results of the pulmonary function test and the oscillometry. Supplementary Table A.2. Differences in Xrs between patients with and without CPFE adjusting for sex, age, %FVC, and %DLCO. [file 12890_2023_2776_MOESM1_ESM.docx]

**Supplementary Material**

Supplementary Table A.1: Baseline characteristics, results of the pulmonary function test and the oscillometry.

|  |  |  | Patients with non–CPFE (n = 148) | | |  | Patients with CPFE (n = 30) | | |  | *p*–value |
| --- | --- | --- | --- | --- | --- | --- | --- | --- | --- | --- | --- |
| Age |  |  | 69 (64 – 75) |  |  | | 69 (65 – 73) | |  | | 0.574 |
| Sex Men / Women | | | 110 / 38 |  |  | | 30 / 0 |  |  | | 0.002^*^ |
| Outcome |  |  |  |  |  | | |  |  | |  |
| Survival / Death / Unknown | | | 58 / 71 / 19 |  |  | | 15 / 10 / 5 |  |  | | 0.340 |
| GAP stage Ⅰ / Ⅱ / Ⅲ | |  | 77 / 53 / 18 |  | | | 16 / 11 / 3 |  |  | | 0.946 |
| BMI | |  | 23.5 (21.6 – 25.9) | |  | | 23.5 (21.3 – 25.5) | |  | | 0.930 |
| Smoking |  |  |  |  |  | | |  |  | |  |
| Current or former / Never | | | 118 / 30 |  |  | | 28 / 2 |  |  | | 0.077 |
| Pack-years | |  | 32 (5–49) |  |  |  | 43 (32 – 67) | |  |  | 0.004^*^ |
| Comorbidities | |  |  |  |  | |  |  |  | |  |
| ^+^ Acute exacerbation of ILD | | | 3 (2.1) |  |  | | 1 (3.4) |  | | | 0.660 |
| Cancer other than lung cancerer | | | 13 (9.6) |  |  | | 4 (15.4) |  | | | 0.439 |
| Hypertension | |  | 52 (54.2) |  | | | 11 (57.9) |  | | | 0.873 |
| Hyperlipidemia | |  | 33 (28.7) |  | | | 7 (30.4) |  | | | 0.901 |
| Diabetes |  | | 32 (27.6) |  | | | 8 (36.4) |  | | | 0.546 |
| Heart diseases | |  | 23 (18.4) |  | | | 4 (15.4) |  | | | 0.759 |
| Arrhythmia | |  | 6 (4.2) |  | | | 1 (3.4) |  | | | 0.853 |
| Pulmonary hypertension | | | 5 (3.5) |  | | | 2 (7.1) |  | | | 0.398 |
| Cerebrovascular diseases | | | 12 (8.8) |  | | | 2 (7.1) |  | | | 0.789 |
| Gastroesophageal reflux | | | 10 (7.2) |  | | | 4 (15.4) |  | | | 0.222 |
| Chronic renal diseases | | | 15 (11.3) |  | | | 2 (7.1) |  | | | 0.556 |
| Bronchial asthma | |  | 8 (5.7) |  |  |  | 2 (7.1) |  |  |  | 0.784 |
| Indices of the PFT | |  |  |  |  |  |  |  |  |  |  |
| VC (L) |  |  | 2.54 (2.13 – 3.12) | |  | | 3.60 (3.20 – 3.79) | |  | | < 0.001^*^ |
| %VC |  |  | 84.8 (70.2 – 99.1) | |  | | 106.3 (84.9 – 118.8) | |  | | < 0.001^*^ |
| FVC (L) |  |  | 2.49 (2.08 – 3.08) | |  | | 3.58 (3.20 – 3.75) | |  | | < 0.001^*^ |
| %FVC |  |  | 83.0 (69.1 – 98.0) | |  | | 106.7 (83.8 – 117.1) | |  | | < 0.001^*^ |
| FEV1 (L) |  |  | 2.06 (1.76 – 2.43) | |  | | 2.73 (2.31 – 3.12) | |  | | < 0.001^*^ |
| FEV1/FVC (%) | |  | 83.4 (78.7 – 88.3) | |  | | 77.5 (69.6 – 80.8) | |  | | < 0.001^*^ |
| ^#^ DLCO (ml/min/mmHg) | | | 11.24 (9.38 – 14.84) | |  | | 11.59 (8.87 – 16.23) | |  | | 0.765 |
| ^#^ %DLCO |  |  | 55.1 (44.8 – 65.8) | |  |  | 55.3 (38.5 – 71.1) | |  |  | 0.720 |
| Indices of the oscillometry | | |  |  |  | |  |  |  | |  |
| R5 (cmH2O/L/s) | |  |  | | | | | | | |  |
| Whole breath | |  | 2.79 (2.31 – 3.42) | |  | | 2.44 (2.04 – 2.72) | |  | | 0.005^*^ |
| Ex |  |  | 2.94 (2.44 – 3.78) | |  | | 2.59 (2.21 – 2.89) | |  | | 0.007^*^ |
| In |  |  | 2.55 (2.10 – 3.13) | |  | | 2.23 (1.83 – 2.57) | |  | | 0.009^*^ |
| ΔR5 |  |  | 0.38 (0.17 – 0.85) | |  |  | 0.33 (0.05 – 0.45) | |  |  | 0.120 |
| R20 (cmH2O/L/s) | |  |  |  |  | |  |  |  | |  |
| Whole breath | |  | 2.16 (1.79 – 2.62) | |  | | 1.90 (1.61 – 2.15) | |  | | 0.002^*^ |
| Ex |  |  | 2.20 (1.83 – 2.82) | |  | | 1.91 (1.67 – 2.11) | |  | | 0.001^*^ |
| In |  |  | 2.01 (1.71 – 2.52) | |  | | 1.77 (1.54 – 2.05) | |  | | 0.004^*^ |
| ΔR20 |  |  | 0.15 (–0.05 – 0.46) | |  |  | 0.08 (–0.07 – 0.16) | |  |  | 0.113 |
| R5–R20 (cmH2O/L/s) | | |  |  |  | |  |  |  | |  |
| Whole breath | |  | 0.63 (0.43 – 0.85) | |  | | 0.53 (0.43 – 0.72) | |  | | 0.110 |
| Ex |  |  | 0.80 (0.48 – 1.03) | |  | | 0.63 (0.49 – 0.86) | |  | | 0.136 |
| In |  |  | 0.48 (0.32 – 0.71) | |  | | 0.46 (0.34 – 0.52) | |  | | 0.200 |
| ΔR5–R20 |  |  | 0.25 (0.09 – 0.45) | |  |  | 0.21 (0.01 – 0.36) | |  |  | 0.199 |
| X5 (cmH2O/L/s) | |  |  |  |  | |  |  |  | |  |
| Whole breath | |  | –0.96 (–1.25 to –0.55) | |  | | –0.69 (–0.80 to –0.23) | | |  | 0.004^*^ |
| Ex |  |  | –0.97 (–1.27 to –0.49) | |  | | –0.67 (–0.82 to –0.28) | | |  | 0.008^*^ |
| In |  |  | –0.88 (–1.20 to –0.54) | |  | | –0.64 (–0.94 to –0.15) | | |  | 0.004^*^ |
| ΔX5 |  |  | 0.06 (–0.13 – 0.22) | |  |  | –0.04 (–0.10 – 0.05) | |  |  | 0.124 |
| Fres (Hz) |  |  |  |  |  | |  |  |  | |  |
| Whole breath | |  | 10.58 (8.17 – 12.41) | |  | | 9.04 (6.55 – 10.5) | |  | | 0.012^*^ |
| Ex |  |  | 10.71 (8.46 – 12.67) | |  | | 8.98 (6.82 – 11.12) | |  | | 0.028^*^ |
| In |  |  | 10.39 (8.40 – 12.23) | |  | | 8.97 (6.18 – 11.01) | |  | | 0.006^*^ |
| ΔFres |  |  | –0.23 (–1.21 – 0.83) | |  |  | –0.06 (–0.24 – 0.50) | |  |  | 0.178 |
| ALX (cmH2O/L/s) | |  |  |  |  | |  |  |  | |  |
| Whole breath | |  | 4.07 (2.18 – 6.19) | |  | | 2.60 (0.76 – 3.39) | |  | | 0.004^*^ |
| Ex |  |  | 4.31 (1.85 – 6.13) | |  | | 2.54 (0.86 – 3.62) | |  | | 0.009^*^ |
| In |  |  | 3.74 (1.96 – 5.93) | |  | | 2.41 (0.50 – 4.21) | |  | | 0.003^*^ |
| ΔALX |  |  | –0.32 (–1.03 – 0.71) | |  |  | 0.16 (–0.25 – 0.41) | |  |  | 0.112 |

Data are presented as median (interquartile range) or number of patients (percentage). ^*^ *p*-value < 0.05, ^+^ Before the first measurement of oscillometry,　^#^ DLCO was measured in 161 cases.

ALX, low-frequency reactance area; BMI, body mass index; CPFE, combined pulmonary fibrosis and emphysema; Δ, difference between expiratory and inspiratory phases; DLCO, diffusing capacity of the lung for carbon monoxide; %DLCO, diffusing capacity of the lung for carbon monoxide (% predicted); Ex, expiratory phase; FEV1, forced expiratory volume in 1 s; Fres, resonant frequency; FVC, forced vital capacity; %FVC, forced vital capacity (% predicted); GAP, Gender–Age–Physiology; ILD, interstitial lung disease; In, inspiratory phase; PFT, pulmonary function test; R5, resistance at 5 Hz; R20, resistance at 20 Hz; R5–R20, difference between R5 and R20; VC, vital capacity; X5, reactance at 5 Hz.

Supplementary Table A.2: Differences in Xrs between patients with and without CPFE adjusting for sex, age, %FVC, and %DLCO.

|  |  | *p*-value |
| --- | --- | --- |
| X5 In |  | 0.692 |
| Fres In |  | 0.932 |
| ALX In |  | 0.588 |

ALX, low-frequency reactance area; CPFE, combined pulmonary fibrosis and emphysema; %DLCO, diffusing capacity of the lung for carbon monoxide (% predicted); Fres, resonant frequency; %FVC, forced vital capacity (% predicted); In, inspiratory phase; X5, reactance at 5 Hz; Xrs, respiratory reactance.
